# Supplementary material for: Mechanical communication-associated cell directional migration and branching connections mediated by calcium channels, integrin β1, and N-cadherin
Source: Front Cell Dev Biol. 2022 Aug 16;10:942058. doi: 10.3389/fcell.2022.942058 (PMC9424768; doi:10.3389/fcell.2022.942058)
Supplement: Supplementary file 3 [file DataSheet1.pdf]

Supplementary information

Mechanical communication-associated cell directional migration and branching connections mediated by calcium channels, integrin β1 and N-cadherin

Mingxing Ouyang[#, *], Yiming Zhu[#], Jiajia Wang, Qingyu Zhang, Yanling Hu, Bing Bu, Jia Guo, Linhong Deng[*]

Institute of Biomedical Engineering and Health Sciences, School of Pharmacy & School of Medicine, Changzhou University, Changzhou City, Jiangsu Province, 213164 China

[#]M.O. and Y.Z. share the first authorships; * corresponding authors.

Supplementary figures:

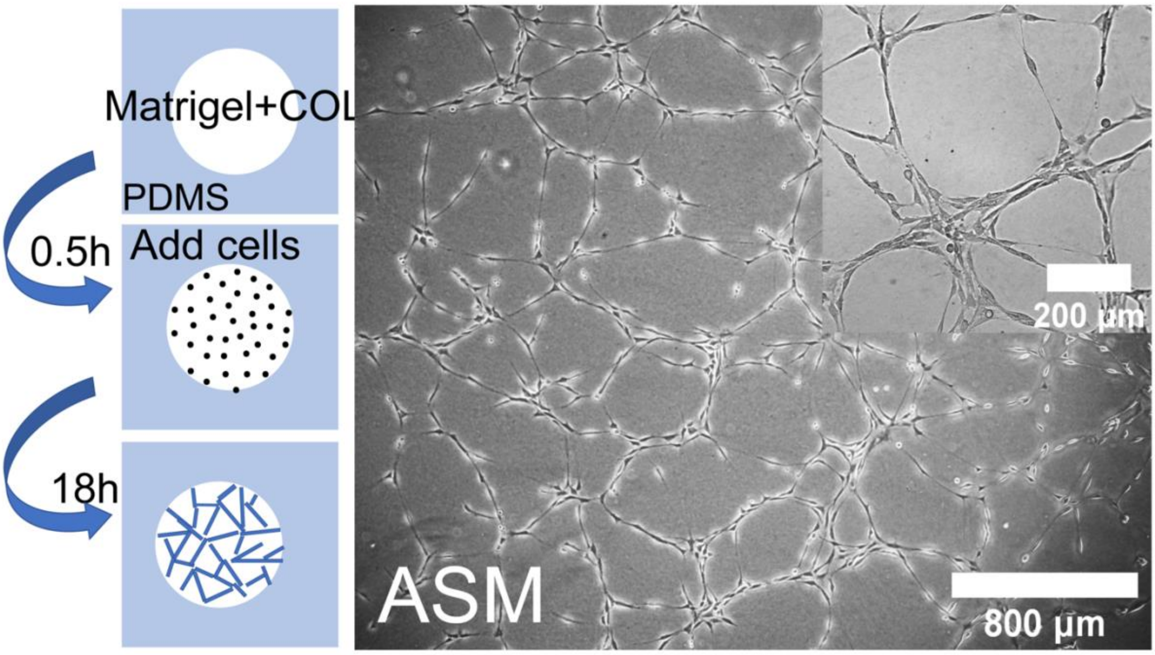

Figure S1. Experimental setup, and the assembled network structures of ASM cells. Cells were seeded on Matrigel hydrogel containing 0.5 mg/ml type I collagen (COL) within the PDMS mold. The images were taken with 5x and 20x objectives under the microscope after 20-h culture.

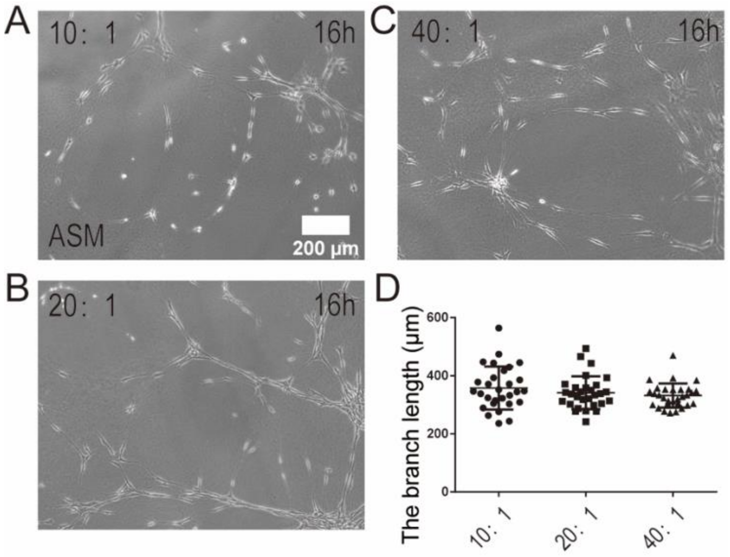

Figure S2. Variable stiffness of PDMS mold boundaries on the branching assembly. The PDMS molds referred in Figure 1A were made with different stiffness by changing the curing agent amount (1/10, 1/20, 1/40 of the base component). (**A-C**) The assembled ASM cell networks after 16 h culture on the hydrogel which was maintained in the PDMS molds with different boundary stiffness as indicated by the curing agent amount. (**D**) The quantified branch lengths under the conditions of (A-C).

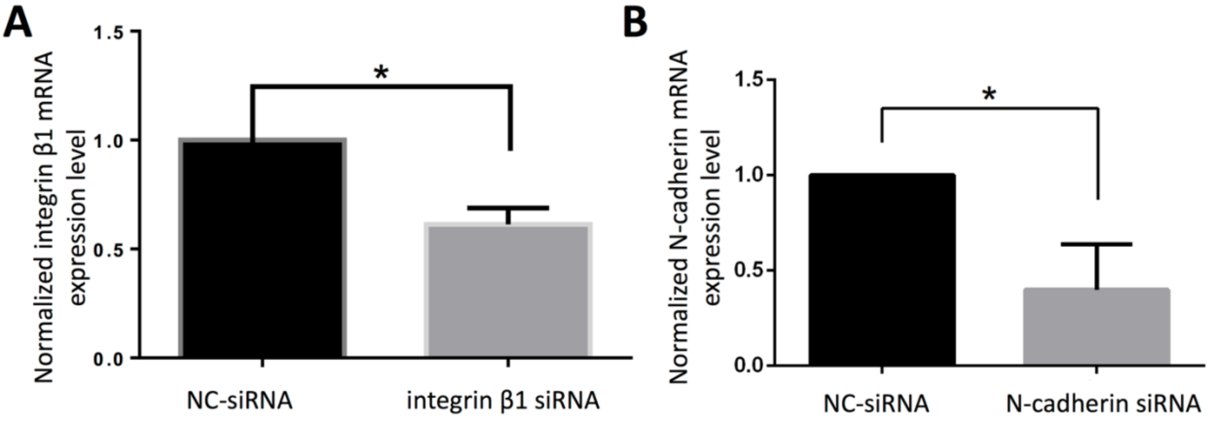

Figure S3. Efficiency of siRNA transfection measured by quantitative PCR (Q-PCR). (**A, B**) ASM cells were transfected with control siRNA (NC) or integrin β1 siRNA (**A**), or N-cadherin siRNA (**B**), and after 72 h, the relative mRNA expression levels were measured by Q-PCR.
